# Oxygen-Dependent Changes in the N-Glycome of Murine Pulmonary Endothelial Cells

**DOI:** 10.3390/antiox10121947

**Published:** 2021-12-04

**Authors:** Akos Tiboldi, Johannes Führer, Wolfgang Schaubmayr, Eva Hunyadi-Gulyas, Marie Louise Zach, Beatrix Hochreiter, Andreas Spittler, Roman Ullrich, Klaus Markstaller, Friedrich Altmann, Klaus Ulrich Klein, Verena Tretter

**Affiliations:** 1 Department of Anesthesia, General Intensive Care and Pain Therapy, Medical University Vienna, 1090 Vienna, Austria; akos.tiboldi@meduniwien.ac.at (A.T.); wolfgang.schaubmayr@meduniwien.ac.at (W.S.); marie.zach@gmx.at (M.L.Z.); trixi.hochreiter@gmx.at (B.H.); roman.ullrich@meduniwien.ac.at (R.U.); klaus.markstaller@meduniwien.ac.at (K.M.); klaus.klein@meduniwien.ac.at (K.U.K.); 2 Division of Biochemistry, Department of Chemistry, University of Natural Resources and Life Sciences, 1090 Vienna, Austria; johannesfue@gmx.at (J.F.); friedrich.altmann@boku.ac.at (F.A.); 3 Laboratory of Proteomics Research, Biological Research Centre, 6726 Szeged, Hungary; gulyas.eva@brc.hu; 4 Department of Surgery and Core Facility Flow Cytometry, Medical University Vienna, 1090 Vienna, Austria; andreas.spittler@meduniwien.ac.at

**Keywords:** lung endothelium, glycocalyx, N-glycosylation, hyperoxia, mass spectrometry

## Abstract

Supplemental oxygen is frequently used together with mechanical ventilation to achieve sufficient blood oxygenation. Despite the undoubted benefits, it is vigorously debated whether too much oxygen can also have unpredicted side-effects. Uncertainty is also due to the fact that the molecular mechanisms are still insufficiently understood. The lung endothelium is covered with an exceptionally broad glycocalyx, carrying N- and O-glycans, proteoglycans, glycolipids and glycosaminoglycans. Glycan structures are not genetically determined but depend on the metabolic state and the expression level and activity of biosynthetic and glycan remodeling enzymes, which can be influenced by oxygen and the redox status of the cell. Altered glycan structures can affect cell interactions and signaling. In this study, we investigated the effect of different oxygen conditions on aspects of the glycobiology of the pulmonary endothelium with an emphasis on N-glycans and terminal sialylation using an in vitro cell culture system. We combined a proteomic approach with N-glycan structure analysis by LC-MS, qRT-PCR, sialic acid analysis and lectin binding to show that constant and intermittent hyperoxia induced time dependent changes in global and surface glycosylation. An siRNA approach identified St6gal1 as being primarily responsible for the early transient increase of α2-6 sialylated structures in response to hyperoxia.

## 1. Introduction

A primary function of the pulmonary endothelium is the formation of a barrier between the blood and the interstitium providing controlled extravasation of fluid, proteins and cells. Inappropriate permeability leads to edema associated with hypoxemia due to impaired gas diffusion and immigration of pro-inflammatory neutrophils as observed in acute lung injury (ALI)/acute respiratory distress syndrome (ARDS) [1]. The pulmonary endothelium is a single cell layer of specialized cells with a unique metabolism, which are connected by tight and adherens junctions to form this barrier [2].

The luminal surface of endothelial cells is covered with a glycan-rich layer, generally termed glycocalyx, which is exceptionally thick (approximately 2 µm) in the lung microvasculature and is even observed with lung microvascular endothelial cells in culture [3]. The glycocalyx comprises glycans from glycoproteins, proteoglycans (syndecans and glypicans) and glycosaminoglycans (heparan sulfate and hyaluronic acid) with a high density of negative charge due to sulfates and sialic acids. The function of the glycocalyx is not fully elucidated, but impaired barrier function frequently goes hand in hand with the shedding of glycocalyx components in ARDS [4].

Degradation of the glycocalyx allows the direct adhesion of neutrophils and facilitates inflammation. While glycosaminoglycans may form the majority of the structure in the glycocalyx, the N- and O-glycans of surface glycoproteins are essential for the function, signal transduction and surface stability of cell surface proteins, such as receptors and cell adhesion molecules. The pulmonary endothelium is in close proximity to the alveoli and, therefore, directly exposed to the inhaled partial pressures of oxygen.

The impact of hypoxia on the glycosylation of proteins has received considerable attention, as changes of glycan structures are frequently observed in hypoxic tumors and the occurrence of special structures often serves as a biomarker for certain types of cancer [5,6]. Hardly explored, however, is the impact of high oxygen concentration (hyperoxia) or intermittent hypoxia/hyperoxia as might occur in atelectatic lungs during ventilation with high oxygen concentrations. Taniguchi et al. coined the term “glyco-redox” to describe the connection between oxidative stress and changes in glycan structure [7].

Evidence has emerged that oxidative stress (which is an imbalance between antioxidants and oxidants, like reactive oxygen species (ROS)) affects transcription factors or epigenetic mechanisms regulating the expression of enzymes in the glycan biosynthetic pathways. The biosynthesis of glycans is also directly influenced by changes in the metabolism of carbohydrates, which is equally redox-sensitive. The resulting changes in glycan structures translate into changes in protein structure and function and might ultimately lead to pathophysiological developments [8].

As the analysis of cell type-specific glycan structures is extremely difficult in vivo, we decided to use an in vitro approach to investigate changes in N-glycan structures of murine lung microvascular endothelial cells in response to different oxygen conditions in the hypoxic/hyperoxic range. Despite the fact that glycosylation might change generally in culture, this model allows the investigation of cell responses to changes in the partial pressure of oxygen, when normoxia is used as the reference condition. We show that N-glycan structures change in response to different oxygen conditions in a time-dependent way, indicating that the partial pressure of oxygen reaching the lung endothelium will have an influence on these cell surface structures, thereby, most likely affecting cellular interactions and signaling.

## 2. Materials and Methods

### 2.1. Cell Culture

C57Bl/6 male mice (age: 6 weeks, Ethical approval: Medical University Vienna and the Federal Ministry for Science, Research and Economy of Austria GZ: BMWFW-66.009/0089-WF/v/3b/2016.) were anesthetized and killed by CO_2_ inhalation. Lungs were excised, homogenized and digested with 0.2% collagenase in HBSS, 1 mM CaCl_2_, 1 mM MgCl_2_ for 45 min in a 37 °C water bath. After the addition of 50 U/mL DNAse I incubation was continued at room temperature for 15 min. Digested tissue was dissociated and filtered through 70 and 40 µm meshes. Endothelial cells were selected by CD31 dynabeads (Thermofisher; Waltham, MA, USA) and plated on gelatin/fibronectin (10 µg/mL)-coated tissue culture plates. CD31 and ICAM1 were used as markers to confirm endothelial identity.

### 2.2. Exposure to Different Oxygen Conditions

Cells were plated into 6-well plates containing a gas-permeable membrane at the bottom (imaging plates, Zellkontakt, Nörten-Hardenberg, Germany) at a density of 2 × 10^5^ cells/well. Direct gas supply was provided from directly underneath the gas-permeable membrane in custom-made exposure boxes. Pre-mixed gas bottles were used to induce hypoxia (5% O_2_, 5% CO_2_, rest N_2_), normoxia (21% O_2_, 5% CO_2_, rest N_2_), severe hyperoxia (95% O_2_, 5% CO_2_) and hypoxic/hyperoxic oxygen oscillations (0–95% O_2_, 5% CO_2_, rest N_2_) by switching electronically between the two bottles every 6 min.

Conditions were chosen to represent O_2_ concentrations that provide a strong stimulus relative to the normoxic control condition without inducing massive cell death for the duration of the experiment. Five percent O_2_ represents relative hypoxia, and 95% O_2_ is a strong hyperoxic condition, both have been shown to trigger typical hypoxic/hyperoxic signaling and changes in gene expression in previous experiments from our research group. The frequency of oscillations in our experiments is smaller compared to what would occur in cyclic atelectasis during mechanical ventilation.

However, a lower frequency is necessary in our setup in order to achieve the full amplitudes on the cellular level, which is limited by the diffusion rate through the gas-permeable membrane of the cell culture plates. This has been previously optimized experimentally. Exposure times represent short-term, intermediate or long-term (chronic) experiments covering a time-frame, where time-dependent changes in glycan structures are observed without any otherwise concomitant influencing factors (such as a decrease in viability).

### 2.3. Semi-Quantitative Proteomic Analysis by Label-Free Liquid Chromatography-Mass Spectrometry (LC-MS)

After exposure to different oxygen conditions cells were lysed with RIPA buffer (25 mM Tris/HCl pH 7.5; 150 mM NaCl; 1% NP-40, 0.5% sodium deoxycholate; 0.1% SDS, protease inhibitor tablet). Proteins were quantified by a bicinchoninic acid-based protein assay, and sample volumes representing 100 µg of total protein amount were precipitated according to the method of Wessel and Fluegge [9]. Proteins were redissolved in 50 µL of 0.5% Rapigest in 50 mM ammonium bicarbonate pH 7.8 and used for analysis.

Disulfide bridges were reduced by addition of 2 µL 100 mM dithiothreitol (DTT) and incubated at 56 °C for 30 min, and free sulfhydryls were alkylated by the addition of 4 μL of 100 mM iodoacetamide (40 min at room temperature in a dark place). Excess alkylation agent was quenched by adding 0.2 µL 100 mM DTT, and in-solution digestion was performed overnight by addition of 2.4 µL of 1 µg/µL trypsin in 50 mM ammonium bicarbonate at 37 °C. Digestion was stopped with 12 µL of 10% formic acid. Each condition was measured in three biological replicates.

Protein identification and semi-quantitative comparison was performed by 2D LC-MSMS analysis. High pH reversed-phase fractionation was performed on a reversed phase column (Phenomenex, Kinetex 5 µm EVO C-18 100 Å, 2.1 × 100 mm) with an elution flow rate of 150 µL/min using gradient elution: 5–40% B in 10 min, 40–95% B in 2 min, 95% B for 3 min 95–5% B in 2 min and 5% B for 7 min (solvent A:10 mM ammonium bicarbonate, pH = 10; solvent B: 10% 10 mM ammonium bicarbonate pH = 10, 90% acetonitrile). We collected 48 fractions from 1 to 25 min (half minute/fraction) and 4–4 fractions were combined (1, 13, 24, 37; 2, 14, 26, 38 etc.) to obtain 12 final fractions.

Each combined fraction was dried down in a vacuum centrifuge, redissolved in 0.1% formic acid/water and subjected to nanoLC-MSMS analysis using an LTQ-Orbitrap Elite (Thermo Scientific, Dreieich, Germany) hybrid mass spectrometer. NanoUPLC runs were performed on a Waters nanoAcquity ULC system (Waters Corp., Milford, CT, USA), using gradient elution after trapping the samples in a trap column (Waters Symmetry C-18, 0.180 mm × 20 mm, 5 µm, 100 Å) with 3% of B (Solvent B: 0.1% formic acid in acetonitrile, solvent A: 0.1% formic acid in water) for 2 min with a 10 µL/min flow rate. The analytical separation was performed with the following gradient: 3–10% B in 5 min, 10–40% B in 32 min. The flow rate was 200 nL/min, and the separating column (Waters BEH130 C-18, 0.075 mm x 250 mm, 1.7 µm, 130 Å) was thermostated to 60 °C.

MS data was acquired in a data-dependent fashion: the 20 most intense multiply charged ions were selected for CID after each survey scan. The survey spectra were measured in the Orbitrap (mass range: 380–1400 *m/z*; resolution: 120,000 @ 400 *m/z*), while the CID MS2 spectra were detected in the ion trap. Selected precursor masses were dynamically excluded for 15 s to facilitate more comprehensive analysis of the sample.

Proteome Discoverer (ver:1.3, Thermo Scientific, Dreieich, Germany) was used to generate MSMS peaklist files and our in-cloud ProteinProspector (ver: 5.18.0) database search engine was used for protein identification. The twelve peaklist files related to one sample were merged and subjected to database search with the following parameters: Database: bovine and mouse sequences from the UniProtKB.2015.4.16. database concatenated with a randomized sequence for each entry (109,150 sequences).

Bovine proteins were considered to account for the fetal calf serum proteins present in the cell culture media. Tryptic peptides with a maximum of one missed cleavage site were considered. Carbamidomethyl cysteine as constant, methionine oxidation, peptide N-terminal pyro-glutamine formation from Gln and protein N-terminal acetylation were set as variable modifications. The error tolerance for the precursor ion was set to 10 ppm and 0.6 Da for fragment masses. A maximum of two variable modifications per peptide was allowed. The ProteinProspector default acceptance criteria were applied: minimum peptide and protein score was set to 15 and 22 respectively.

Pairwise semi-quantitative analysis was performed in an excel table: The calculated false discovery rates (FDR) were less than 1% in all cases, and two further criteria have to be fulfilled to consider a protein-hit real. At least in one of the groups of protein has to be identified the protein from all three replicates and at least five unique peptides have to match the protein in two cases from the three replicates. The base of our semi quantitative analysis is the number of the spectra identifying the particular protein (peptide count) divided by the total number of PSM (peptide spectrum match) of the sample.

To avoid the calculation problem when a protein shows a spectral count zero in one of the samples, the following formula was used to calculate the relative spectral counts: RPC = (n + f)/(t + f), where RPC is the relative peptide count, n is the number of spectra identified for the protein, t is the total number of identified spectra in the sample, and f is a correction factor, set to 1. A *t*-test was performed (two-sample Student’s *t*-test with unequal variances) to decide whether the difference between any two groups (with three replicates) was significant (*p* < 0.05). The averages of the relative peptide counts were compared and at least two-fold changes were considered as real difference. It was assumed that most proteins do not show quantitative changes, therefore, relative peptide count ratios were normalized to the median values.

### 2.4. Cell-Surface Lectin Binding Analysis

Cells were detached by treatment with Accutase (Sigma-Aldrich, St. Louis, MO, USA), fixed with 2% formalin for 15 min at room temperature and centrifuged. The pellet was resuspended in 1 mL PBS and incubated with FITC-labeled *Sambucus nigra* (SNA) or *Maackia amurensis* (MAL I) lectin (all from Vector Laboratories, Burlingame, CA, USA) in a 1:200 dilution. After 20 min incubation at room temperature, cells were washed twice with PBS and analyzed by flow cytometry on a Beckman Coulter CytoFLEX LX. The read-out unit was the mean fluorescence intensity (MFI). In order to visualize treatment-dependent changes in surface sialylation, MFI values were normalized as the fold change relative to the cells exposed to 21% O_2_ for the same period of time.

### 2.5. Quantification of Sialic Acids

Sialic acids (N-acetylneuraminic acid and N-glycolylneuraminic acid) were derivatized by reaction with 1,2-diamino-4,5-methylenedioxybenzene (DMB) according to the method described by Hara et al. [10] Briefly, samples were hydrolyzed in 2 M acetic acid at 80 °C for 2.5 h and dried and incubated with DMB (Sigma-Aldrich, St. Louis, MO, USA) at 50 °C for 2.5 h in the dark.

The resulting fluorescent products (quinoxalinones) were separated by High Performance Liquid Chromatography (HPLC) [(Nexera X2 HPLC system; column: Hypersil-Keystone ODS (250 × 4 mm, 5 μm particle size; Thermo Scientific, Vienna, Austria); flow rate 1 mL/min, with a linear gradient from 30% to 42% Eluent B (70% 100 mM NH_4_CH_3_COO pH 5.5, 30% acetonitrile) in 12 min; Eluent A (water)]. Fluorescent products were detected in a flow-through fluorimeter (Ex/Em = 373 nm/448 nm) with a RF-20Axs Fluorescence Detector, semimicro flow cell (Shimadzu, Korneuburg, Austria); Standard N-Acetylneuraminic acid was purchased from Sigma-Aldrich, St. Louis, MO, USA, N-Glycolylneuraminic acid from Calbiochem, San Diego, CA, USA.

### 2.6. Quantitative Analysis of N-Glycan Structures

Adherent cells were lysed using 100 mM ammonium bicarbonate with 1% SDS. In order to denature the three-dimensional structure of proteins and to facilitate deglycosylation, dithiothreitol was added to a concentration of 30 mM, and the sample was incubated for 5 min at 95 °C followed by 30 min at 56 °C, then iodoacetamide was added to a concentration of 75 mM and incubated for 30 min in the dark at room temperature. After centrifugation at 10,000 rcf for 5 min, proteins from the supernatant were precipitated using chloroform/methanol [8]. After redissolving proteins in 50 mM ammonium acetate buffer (pH 8.4), N-glycans were released by a PNGase F treatment (16 h at 37 °C).

The reaction was stopped with 800 mM ammonium/formate buffer (pH 3.0) and 7.25 pmol of ^13^C_6_-labelled disialylated diantennary N-glycan (NaNa), which served as retention time corrective, was added. Released glycans were separated from proteins with C_18_ solid phase extraction cartridges (Thermo Scientific, Waltham, MA, USA) according to the manufacturer instruction. Glycans from the flowthrough were reduced with 1% NaBH_4_ in 50 mM NaOH. The reduced glycans were purified with porous graphitized carbon (PGC) solid phase extraction cartridges (Thermo Scientific, Waltham, MA, USA) according to the manufacturer’s protocol. Glycans were eluted with 55% acetonitrile in ammonium formate (pH 3.0), dried and taken up in HQ-H_2_O.

#### Liquid Chromatography-Mass Spectrometry (LC-MS)

Purified samples were loaded on a PGC column (100 × 0.32 mm, 5 μm; Thermo Scientific, Waltham, MA, USA) using 80 mM ammonium formate buffer of pH 3.0 as the aqueous solvent (solvent A). A linear gradient from 1% solvent B (80% acetonitrile plus 20% solvent A) to 65% solvent B in 39 min was applied, at a flow rate of 6 μL/min.

All samples were measured in positive mode with a quadrupole time-of-flight (Q-TOF) instrument (maXis 4G; Bruker, Bremen, Germany) equipped with the standard ESI source in data-dependent acquisition mode (switching to MS/MS mode for eluted peaks), directly linked to the Thermo Ultimate 3000 UPLC system. Standard source settings (capillary voltage 4.5 kV, nebulizer gas pressure 0.5 bar, drying gas 5 L/min, 200 °C) were used. Instrument tuning was optimized for a mass range between 150–2200 Da. Instrument calibration was performed with an ESI calibration mixture (Agilent, Santa Clara, CA, USA).

Data interpretation was performed with DataAnalysis 4.0 and QuantAnalysis 2.0 (Bruker, Bremen, Germany). First, all glycans present in each sample type were determined in DataAnalysis 4.0. Afterwards, an automated script was created in QuantAnalysis 2.0, which searches an extracted ion chromatogram (XIC) of each glycan for a peak at the given retention time. For the calculation of each XIC, the first four isotopic peaks of all charge states within the mass range as well as all ammonium and sodium adducts found in the sample were taken into consideration. In the case of overlapping peaks, less isotopic peaks were used to avoid errors.

### 2.7. Quantitative Real-Time PCR

mRNA was isolated using the RNeasy kit (Qiagen, Hilden, Germany) from an individual confluent well, dissolved in 30 µL RNase-free water and quantified by reading the OD at 260 nm with a Nanodrop 2000 (Thermo Scientific, Waltham, MA, USA). 0.5–1.0 µg mRNA were reverse transcribed using the qScript master mix (Quanta Biosciences, Beverly, MA, USA). cDNA was analyzed on a Rotor Gene Q cycler (Qiagen, Hilden, Germany) using Perfecta SYBR Green Fast mix (Quanta Biosciences, Beverly, MA, USA) and the primers listed in Table 1. The temperature program comprised 30 s at 95 °C for denaturing and 45 cycles of 5 s at 95 °C, 15 s at 55 °C and 10 s at 72 °C. Beta-actin was used for internal normalization.

The following primer sequences were used to target glycosyltransferases (see Abbreviations section):

**Table 1:** Sequences of the primers used for qPCR.

Primers	Sequences
***NANS.***	5′-CAC CAA GGA GAC ATA GAT GTG GC-3′
	5′-CTC TTC TGA AAC TTA GCG CAG T-3′
***St6gal1.***	5′-CTC CTG TTT GCC ATC ATC TGC-3′
	5′-GGG TCT TGT TTG CTG TTG AGA-3′
***St3gal1.***	5′-GGA GAG AAT GTC AAC ATG GTC C-3′
	5′-GTG TGA GTG ATG GTG CCT GT-3′
***St3gal2.***	5′-TTC GGG TGT GGT TCC TCT CTA-3′
	5′-CGC TGT AGT CCT GAA TAG CCT-3′
***St3gal3*.**	5′-AAG CTG GAC TCT AAA CTG CCT-3′
	5′-TGC TGG CTT GGA GAA CCT G-3′
***OGT.***	5′-GCA GCA ACC AAA CTT TGC AGT-3′
	5′-TCA AGG GTG ACA GCC TTT TCA-3′
***GFAT1.***	5′-GAA GCC AAC GCC TGC AAA ATC-3′
	5′-CCA ACG GGT ATG AGC TAT TCC-3′
***St3gal5.***	5′-ATG CCA AGT GAG TTC ACC TCT-3′
	5′-ACT CCA AAT GCA ACC AAC GTG-3′
***St3gal6.***	5′-ACT GTG GGG AAC AAA TGG CTA-3′
	5′-GGA CAT GGC AGC AAC CTT T-3′
***FUT3.***	5`-CCT CCC GGT GTT GCA TA-3′
	5′-CCG TGA AAC GCA CAT AC-3′
***FUT8.***	5′-GGT TCC TGG CGT TGG ATT ATT-3′
	5′-TCA ATG GGG CCT TCT GGT ATT-3′
***Actb.***	5′-AGC CAT GTA CGT AGC CAT C-3′
	5′-CTC TCA GCT GTG GTG GTG A-3′
***UAP1.***	5′-ACT CCC AGG GGC ACT TCA TTA-3′
	5′-GGC CTC TGA CTT TCC ATT TGT3′

### 2.8. siRNA Induced Knock-Down of Sialyl Transferases

Cells were plated into 6-well culture plates and grown until 80% confluence. Transfection mixes were prepared: Solution A: 80 pMol of St6gal1 or St3gal6 siRNA (Ambion, Austin, TX, USA) diluted in 100 µL Opti-MEM (GIBCO, ThermoFisher, Waltham, MA, USA); Solution B: 5 µL siRNA transfection reagent (Santa Cruz, Dallas, TX, USA) in 100 µL Opti-MEM. Both solutions were mixed, incubated at room temperature for 45 min and then diluted with 800 µL Opti-MEM and added to prewashed cells.

After an overnight incubation, the medium was replaced with growth medium containing 20% fetal calf serum and cells were further incubated for 24 h before exposure to different oxygen conditions. At 40 h after transfection, the knockdown efficiency was determined by Western blot analysis of cell lysates from wildtype and St6gal1 or St3gal6 siRNA treated cells. Antibodies were from Sigma (Burlington, MA, USA) and NOVUS Biologicals (Englewood, CO, USA) respectively. The protein levels were reduced by 75% in St6gal1 knockdowns and by 82% in St3gal6 knockdowns (see Supplemental Data S3).

### 2.9. Statistical Analysis

Data were obtained from three independent cell preparations. For quantifications of N-glycan structures at baseline (=0 h), values are given as the mean ± standard deviation. Exposure to different oxygen conditions was performed with *n* = 3 cell preparations for 4 and 24 h, respectively. Data from these three cell preparations are depicted in graphs of glycan structures as individual values and the horizontal line represents mean values.

qRT-PCRs were performed from two cell preparations in *n* = three replicates each with similar results. Data in graphs represent the mean fold change ± standard deviation relative to control conditions (21% O_2_) from one experiment (*n* = 3 individual wells). Testing for statistical significance was done using Student’s *t*-test comparing individual fold changes to control condition, or one-way ANOVA to compare more groups with *p* = 0.05 as the threshold.

## 3. Results

### 3.1. Experimental Workflow

**Figure 1:**
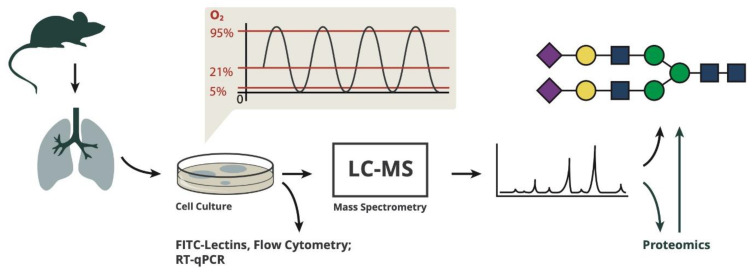
The procedures and analyses performed in this study.

Primary endothelial cells were prepared from C57Bl/6 mouse lungs in a total of three independent cell preparations (performed in January, May and August 2019). After isolation from the lungs, the cells were put into culture and expanded for 3 weeks to have sufficient cell mass for analysis. During the culture period, cells were repurified by magnetic separation in order to select for cells with endothelial surface markers, to minimize contamination with other cell types like fibroblasts and to remove cells that lost the endothelial phenotype during culture. After 3 weeks, cells were repurified via magnetic separation with anti-CD31 beads before being plated into special culture plates with gas-permeable bottom membranes and were exposed to different gas conditions for 4, 24 and 72 h (Figure 1).

### 3.2. High Oxygen Affects Expression of Enzymes Involved in Glycan Biosynthesis

We performed semi-quantitative label-free proteomic analysis from cells exposed to strong constant and intermittent hyperoxia and found that protein expression of three key enzymes in glycan biosynthetic pathways are altered after 24 and 72 h of exposure to strong or intermittent hyperoxia (Table 2).

**Figure 2:**
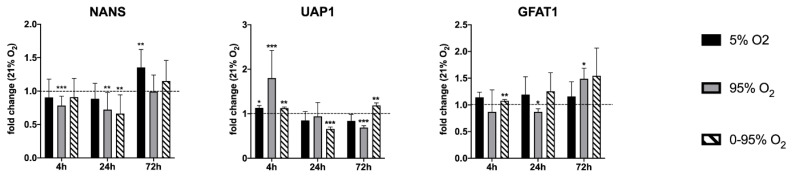
Expression of mRNA levels of NANS, UAP1 and GFAT1 as determined by RT-qPCR. Values are given as the fold change relative to control condition (21% O_2_) at the same time point. * *p* < 0.05, ** *p* < 0.01 and *** *p* < 0.001.

The 24 h intermittent hypoxia/hyperoxia (0–95% O_2_) decreased the expression of glutamine-fructose-6-phosphate-aminotransferase 1 (GFAT1), the rate-limiting enzyme of the hexosamine pathway and sialic acid synthase (NANS) to one third. Long-term (72 h) constant hyperoxia (95% O_2_) and intermittent hypoxia/hyperoxia (0–95% O_2_) led to an upregulation of UDP-N-acetylhexosamine-pyrophosphorylase-like-protein-1 (UAP1) (fold change compared to 21% O_2_ = 2.2 and 2.3 respectively). The mRNA levels of these enzymes were quantified by RT-qPCR (Figure 2).

### 3.3. Sialic Acid Specific Lectin Binding to Cell Surface Glycans

**Figure 3:**
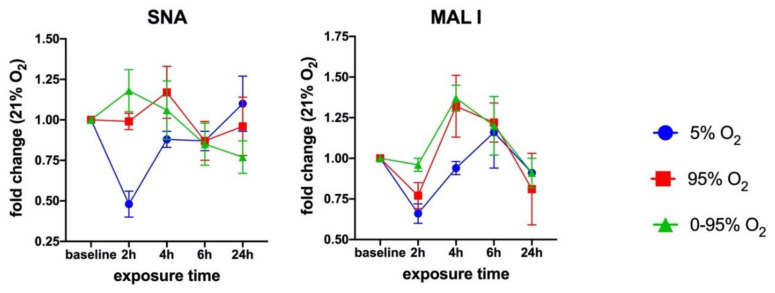
Time-dependent cell surface binding of *Sambucus nigra* agglutin (SNA) and *Maackia amurensis* I (MAL I) lectin under moderate hypoxia (5% O_2_), strong constant hyperoxia (95% O_2_) and intermittent hypoxia/hyperoxia (0–95% O_2_). Values from flow cytometry data are given as the mean ± SD of the fold change of the Mean Fluorescence Intensities (MFI) relative to 21% O_2_.

In order to assess the overall cell surface sialylation, we used lectin labeling with FITC-SNA (with preference for αNeu5Ac-(2-6)-Gal or αNeu5Ac-(2-6)-GalNAc) and FITC-MAL I (αNeu5Ac-(2-3) Gal ß1-4GlcNAc) and analyzed fixed cells by flow cytometry. These epitopes can occur on N- and O-glycans as well as glycolipids. Both lectins revealed O_2_-dependent changes over a period of 24 h (Figure 3).

### 3.4. Analysis of Time-Dependent Changes in N-Glycan Structures

We analyzed samples at 4, 24 and 72 h. Results from 72 h were mostly indistinguishable from the baseline (=0 h) values; therefore, these data are not shown in the graphs. All quantitative calculations were done by comparing O_2_-condition dependent changes relative to the control condition (=21% O_2_) of the same cell preparation at the same time point.

**Figure 4:**
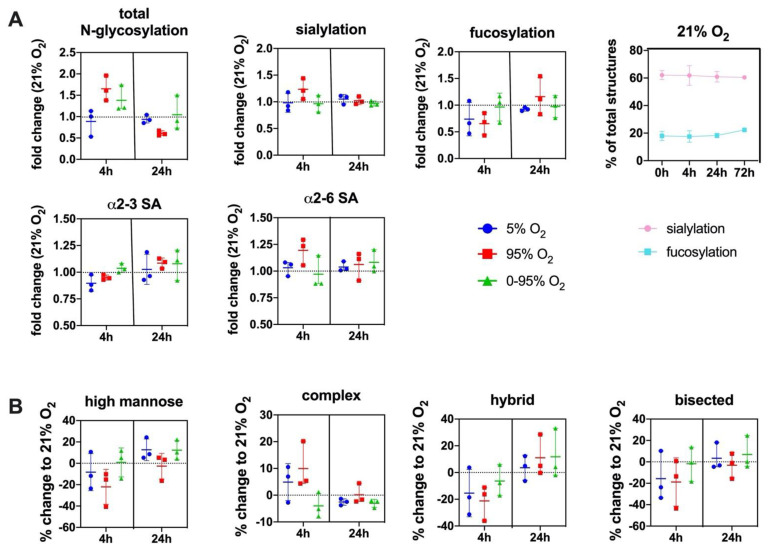
(**A**) Changes in the overall N-glycosylation, sialylation and fucosylation of N-glycans under different O_2_ conditions over time. SA = sialic acid. Top right panel: Change of overall sialylated and fucosylated N-glycans as the % of total N-glycans over the duration of the experiments (0–72 h) under normoxic (21% O_2_) conditions. (**B**) Change in abundance of different N-glycan types over time under different O_2_ conditions relative to control condition (21% O_2_). Values are given as individual measurements (*n* = 3) including the mean ± range or mean ± SD (top right panel): (**A**): fold change relative to 21% O_2_ or% of total N-glycan structures (**B**) % change relative to 21% O_2_.

This is necessary to compensate for changes in glycosylation that are due to variability that is caused by the use of primary cell cultures from different animals (individual variability) and by de-differentiation tendencies of mouse lung endothelial cells during culture. Changes of the overall sialylated and fucosylated N-glycans as parts of the total N-glycan structures over the duration of the experiments (0–72 h) under normoxic conditions (21% O_2_) and variability between different experiments is depicted in Figure 4A (top right panel).

The total degree of N-glycosylation varied between different oxygen conditions depending on the exposure time. The fold changes compared to 21% O_2_ at the same time point were: 4 h 5% O_2_ (0.88 ± 0.30; n.s.); 4 h 95% O_2_ (1.65 ± 0.29; *p* = 0.018); 4 h 0–95% O_2_ (1.38 ± 0.31; n.s.); 24 h 5% O_2_ (0.95 ± 0.13; n.s.); 24 h 95% O_2_ (0.62 ± 0.08; *p* = 0.026); and 24 h 0–95% O_2_ (1.12 ± 0.53; n.s.) (Figure 4A).

At baseline, our method identified 67 different N-glycan mass levels comprising a total of 151 structures (see Supplemental Data S2). The predominant type of glycans were complex glycans (72.5 ± 4.5% at baseline), followed by high-mannose (19.22 ± 4.10% at baseline), hybrid (8.23 ± 0.62% at baseline) and bisected glycans (2.66 ± 1.24% at baseline). The most abundant structure was the trisialylated tri-antennary complex glycan (NaNaNa: 44.24 ± 7.98%). 66.07 ± 6.22% of all structures were sialylated (one sialic acid: 7.92 ± 1.15%; two sialic acids: 13.90 ± 0.72%; three sialic acids: 44.24 ± 7.98% NaNaNa; α2-3/α2-6 linkage ratio at baseline = 1.06 ± 0.24), and 16.60 ± 3.05% of all structures were fucosylated (one fucose: 13.60 ± 2.13%; two fucoses: 2.98 ± 1.20%).

The total sialylation exhibited a maximum at 4 h constant hyperoxia (fold change relative to 21% O_2_: 1.23 ± 0.19) with an increase of sialic acids in α2-6 linkage (fold change: 1.17 ± 0.17 relative to 21% O_2_). Sialic acids in α2-3 linkage showed a slight increase later at 24 h hyperoxia (1.08 ± 0.14 relative to 21% O_2_). Some of the higher and more complex sialylated structures (including mixed linkages) could not be unambiguously individually identified by their retention time in our setting.

Therefore, these structures are not included in the quantifications of α2-3 or α2-6 sialylated glycans. Total fucosylation showed a decrease at 4 h hyperoxia (fold change to 21% O_2_: 0.65 ± 0.21) and only later, an increase at 24 h hyperoxia (fold change to 21% O_2_: 1.16 ± 0.36) (Figure 4A). In order to verify our finding that, otherwise in mouse lungs, highly expressed N-glycolylneuraminic acid (Neu5Gc) only occurs in minor abundance in lung endothelial cells, we hydrolysed all sialic acids, derivatized with DMB and analyzed by HPLC. These data confirmed that Neu5Gc only comprised around 3% of all sialic acids in mouse endothelial cells (data not shown).

Constant hyperoxia and hypoxia showed a parallel development over time regarding the type of N-glycan, although the effect was more pronounced under severe hyperoxia. In these groups complex structures reveal a maximum at 4 h (mean values: hyperoxia: +9.9% and hypoxia: +4.9%), while high mannose (mean values: hyperoxia: −22.1% and hypoxia: −8.3%) hybrid (mean values: hyperoxia: −21.3% and hypoxia: −15.4%) and bisected (mean values: hyperoxia: −18.8% and hypoxia: −15.7%) structures decrease. Hypoxic/hyperoxic oscillations had a different trend: the mean values for complex N-glycans (−4.0%); high mannose (+0.8%); hybrid (−6.4%); and bisected (−1.8%) (Figure 4B).

The antennarity of a glycan is determined by the number of N-acetylglucosamine residues attached to the core N-glycan structure. Most glycans at baseline were found to be tri-antennary (52.13 ± 8.65%), followed by di-antennary (19.15 ± 4.34%) and tetra-antennary structures (1.24 ± 0.16%). Under constant hypoxia, around 10% of di-antennary N-glycans shifted towards tri-antennary glycans at 4 h, an effect that is even less pronounced under hyperoxia. All three conditions led to a decrease of tetra-antennary structures from 4 to 24 h, and, after that, the curve returned to baseline (data not shown).

**Figure 5:**
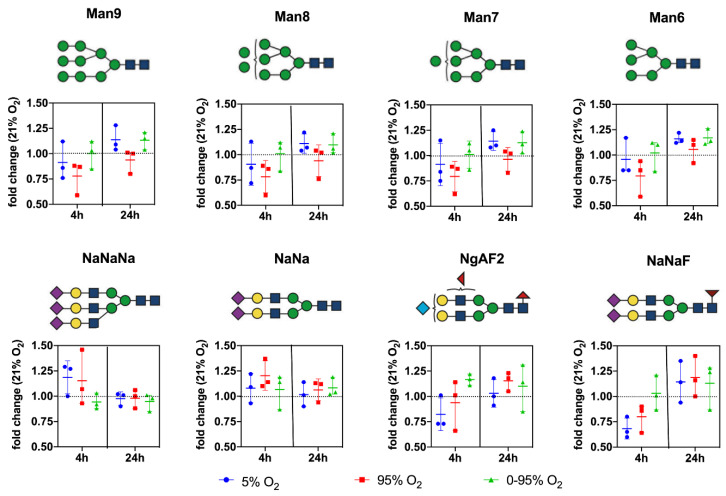
Changes in the relative abundance of individual prominent high mannose and complex type N-glycan structures under different O_2_ conditions over time. Values are given as individual measurements (*n* = 3) including the mean ± range (fold change relative to 21% O_2_).

At baseline, the following structures with a prevalence of >0.1% of total glycans were quantified (given as% mean ± SD; *n* = 3) (for explanations on abbreviations see Supplemental Information S1A and S1B): High Mannose and Hybrid N-glycans: Man9 (5.83 ± 1.44); Man9Glc (0.88 ± 0.35); Man8 (5.35 ± 1.34); Man7 (2.54 ± 0.54); Man6 (2.36 ± 0.44); Man5 (1.61 ± 0.33); Man5Gn (0.36 ± 0.25); Man5Gn + 1Hex (0.49 ± 0.20); Man5Na (0.82 ± 0.25); Man5Ng (0.14 ± 0.01); Man5GnF (0.20 ± 0.08); Man4 (0.63 ± 0.09); Man4Na (0.69 ± 0.25); MM (0.70 ± 0.04); MMF (0.24 ± 0.06); and GnGnGnF (0.55 ± 0.23).

(Sialylated) complex and bisected N-glycans: NaNaNa (44.24 ± 8.0); NaNa (5.89 ± 1.09); NaNaA (1.77 ± 0.42); NgAF2 (2.86 ± 1.19); NaNaF (2.15 ± 0.73); NaAF (1.61 ± 0.50); NaAAAF (0.35 ± 0.14); NaGnF (0.38 ± 0.05); NgA (0.12 ± 0.08); NaGn (0.22 ± 0.05); NaNaAF (1.87 ± 0.50); NaMan5bi (1.26 ± 0.57); NaA (1.77 ± 0.06); NaAAA (0.17 ± 0.06); NaM (0.17 ± 0.03); and NaMF (0.14 ± 0.05). Abundant representatives and their changes under different oxygen conditions over time are depicted in Figure 5.

From the graphs, it is evident that hyperoxic and, to a lesser extent, also hypoxic conditions led to a decrease of high mannose glycans at 4 h returning to baseline levels thereafter. In contrast, high mannose glycans remained constant over the whole time period under intermittent hypoxia/hyperoxia.

The complex sialylated structures NaNaNa and NaNa increased at 4 h under constant hyperoxic and hypoxic conditions and returned to baseline after that. Fucosylated structures (represented by NaNaF and NgAF_2_ in Figure 5) showed a trend to increase only at 24 h under hyperoxic conditions.

### 3.5. Analysis of Time-Dependent Changes in mRNA Expression of Sialyl-And Fucosyl Transferases

**Figure 6:**
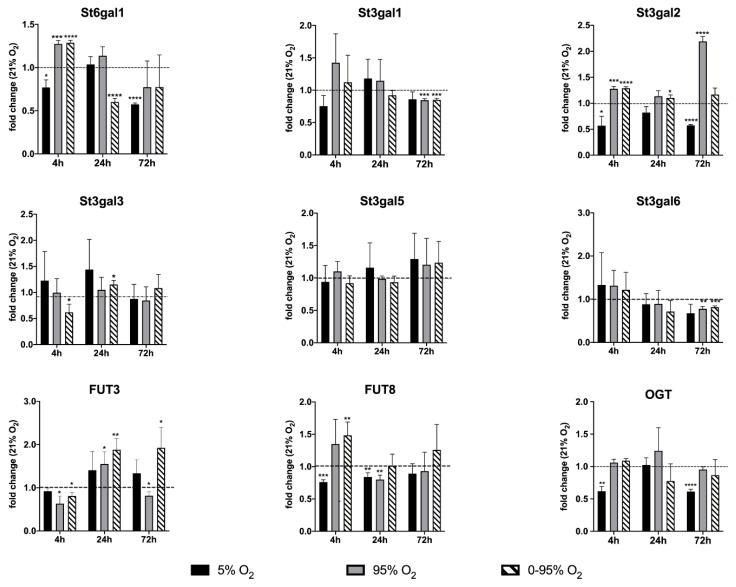
Expression analysis of glycosyl transferase mRNA levels by qRT-PCR. Values are given as the fold change relative to the control condition (normoxia: 21% O_2_). For statistical analysis, Student’s *t*-test was used to compare each group to the control group. * *p* < 0.05; ** *p* < 0.01; *** *p* < 0.001; and **** *p* < 0.0001.

Using qRT-PCR (see Figure 2 and Figure 6), we confirmed the down-regulation of mRNA levels of NANS under constant hyperoxia after 4 h (fold change relative to 21% O_2_: 0.78 ± 0.14; *p* = 0.0007) and 24 h (fold change relative to 21% O_2_: 0.72 ± 0.26; *p* = 0.079) and under hypoxic/hyperoxic oscillations at 24 h (0.66 ± 0.28; *p* = 0.0051). At 72 h NANS was upregulated under constant hypoxia (fold change relative to 21% O_2_: 1.35 ± 0.27; *p* = 0.0028). N-glycans are sialylated by sialyl transferases of the St6gal and St3gal family exemplified by St6gal1 and St3gal6.

St6gal1 was down-regulated under 4 and 72 h constant hypoxia (fold change: 0.77 ± 0.09; *p* = 0.0103 and 0.57 ± 0.02, *p* < 0.0001, respectively) and 24 h hypoxic/hyperoxic oscillations (0.60 ± 0.04; *p* < 0.0001), but up-regulated after 4 h constant hyperoxia (1.27 ± 0.04; *p* = 0.0003) and hypoxic/hyperoxic oscillations (1.28 ± 0.03; *p* < 0.0001). St3gal6 showed a tendency of upregulation at 4 h of hyperoxia and hypoxic/hyperoxic oscillations (though not statistically significant) and was down-regulated at 72 h constant hyperoxia (0.77 ± 0.06; *p* = 0.0024) and hypoxic/hyperoxic oscillations (0.82 ± 0.03; *p* = 0.0004).

Some insight into changes in sialylation of other than N-glycans provide expression levels of other St3gal family members. After an early tendency of upregulation under hyperoxia, St3gal1 was down-regulated at 72 h under constant and intermittent hyperoxia (0.85 ± 0.023; *p* = 0.0003, and 0.85 ± 0.03; *p* = 0.0006). St3gal2 was upregulated at 4 h constant hyperoxia (1.27 ± 0.05; *p* = 0.015) and intermittent hypoxia/hyperoxia (1.28 ± 0.03, *p* < 0.0001), but down-regulated at 4 and 72 h hypoxia (0.56 ± 0.18; *p* = 0.015 and 0.57 ± 0.02; *p* < 0.0001). St3gal5 showed no significant change under any condition and at all time points.

A functionally important modification of glycans is fucosylation, which occurs either as “core-fucosylation” of the asparagine-linked innermost N-acetyl-glucosamine (in mammals in α1-6 linkage), or as outer capping structure linked to terminal galactose (in α1-2 linkage) or sub-terminal N-acetyl-glucosamine (in α1-3 and α1-4 linkages).

We looked at two key fucosyl transferases: FUT8 (core-fucosylation of N-glycans) and FUT3 (an α1-3 and α1-4 fucosyl transferase involved, among others, in the creation of the sialyl-Lewis A and sialyl-Lewis X epitope on N- and O-glycans). FUT8 was down-regulated at 4 h constant hypoxia (0.76 ± 0.04; *p* = 0.0001) but up-regulated under hypoxic/hyperoxic oscillations (1.48 ± 0.21; *p* = 0.015). At 24 h, FUT8 was still down-regulated under constant hypoxia (0.84 ± 0.06; *p* = 0.0063) and also down-regulated under constant hyperoxia (0.80 ± 0.07; *p* = 0.0069). FUT3 was down-regulated after 4 h constant hyperoxia (0.63 ± 0.18; *p* = 0.0137) and hypoxic/hyperoxic oscillations (0.81 ± 0.083; *p* = 0.0112).

At 24 h, both conditions turned into up-regulation of FUT3 (constant hyperoxia: 1.55 ± 0.28; *p* = 0.020; hypoxic/hyperoxic oscillation: 1.88 ± 0.26; *p* = 0.004). At 72 h, hypoxic/hyperoxic oscillations FUT3 was up-regulated (1.93 ± 0.48; *p* = 0.0221), while, under constant hyperoxia, FUT3 was down-regulated (0.82 ± 0.09; *p* = 0.0268). O-linked N-acetylglucosamine transferase (OGT) has a special role in linking a single GlcNAc to a serine or threonine and can, thereby, influence signaling by phosphorylation, activity of transcription factors and degradation via the proteasome. The expression of OGT mRNA was decreased after 4 and 72 h exposure to constant hypoxia (4 h: 0.618 ± 0.074; *p* = 0.0022; 72 h: 0.613 ± 0.038; *p* < 0.0001).

**Figure 7:**
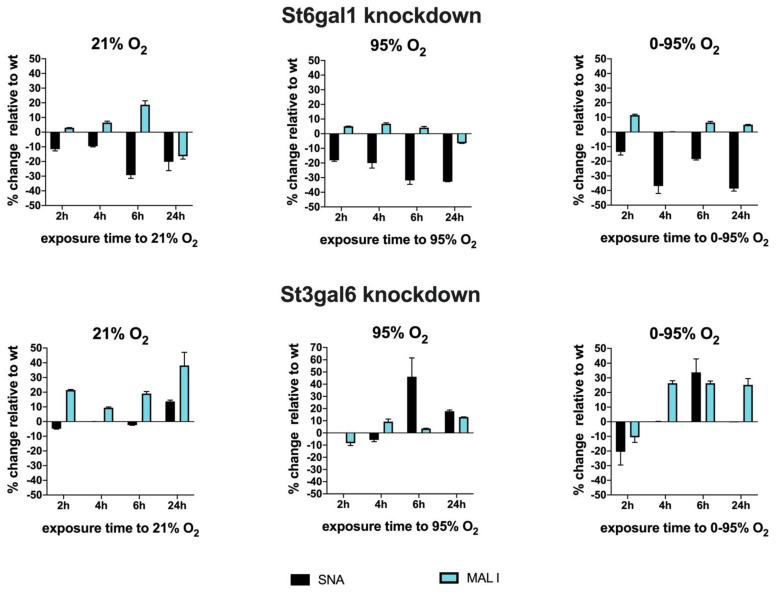
siRNA knockdown of St6gal1 and St3gal6 sialyl transferases and analysis of cell surface binding of lectins SNA and MAL I. Exposure to different oxygen conditions started 36 h after transfection with siRNAs. Values are given as% change of lectin binding (MFI) relative to sham transfected cells. Wildtype (wt), (mean ± SD).

To investigate the dynamics of sialyl transferase expression and activities, we performed siRNA knockdown experiments of two transferases as examples of the representatives involved in α2-6 and α2-3 sialylation, i.e., St6gal1 and St3gal6 (Figure 7).

At 36 h after transfection of siRNAs or sham transfection as control, we started exposure to 21% O_2_, 95% O_2_ or 0–95% O_2_ oscillations to investigate sialylation dynamics under hyperoxic conditions. It is necessary to point out that changes in overall surface sialylation will show with significant time delay, as these structures have been inserted prior to knockdown, and their turnover depends on glycoprotein turnover and might be slow. However, knocking down sialyl transferases inhibits their activity towards newly synthesized glycoproteins and also modifies the activity of other sialyl transferases that are present in the cell.

St6gal1 knockdowns revealed a significant reduction up to −29.3 ± 2.2% (6 h exposure) of SNA lectin binding that was slightly further enhanced to −32.7 ± 0.02% (24 h exposure) under 95% O_2_ and −38.7 ± 1.7% (24 h exposure) under 0–95% O_2_. The loss of SNA binding was only slightly compensated by upregulation of MAL I binding under 95% O_2_ (max. +6.8 ± 0.6% at 4 h exposure) and 0–95% O_2_ (max. +11.6 ± 0.6% at 2 h exposure). St3gal6 knockdowns however reveal obviously some compensation by another sialyl transferase that can synthesize glycans with sialic acids in α2-3 configuration.

MAL I surface binding was therefore upregulated up to +38.3 ± 8.8% (after 24 h exposure) under 21% O_2_. SNA binding remained more or less unaltered. However, exposure to constant or intermittent hyperoxia resulted in an upregulation of SNA binding of up to +46.2 ± 15.4% (6 h exposure to 95% O_2_) and to +33.8 ± 9.2% (6 h exposure to 0–95% O_2_) and decreased again thereafter. This peak correlates, despite a small temporal delay, to the observation of an increase of α2-6 sialylated N-glycan structures at 4–6 h exposure to hyperoxic conditions and is absent in the St6gal1 knockdowns, therefore, implicating that St6gal1 is responsible for the increase of α2-6 glycan structures at 4–6 h of exposure to hyperoxia.

## 4. Discussion

In this study, we aimed to investigate whether altered partial pressures of oxygen, as they are encountered directly by the pulmonary endothelium in vivo, have an influence on proteins involved in the synthesis and modification of cellular glycan structures in pulmonary endothelial cell cultures. While the effects of oxidative stress and derived ROS on O-linked glycans and glycosaminoglycans has received considerable attention in the literature, impact on N-glycosylation is much less explored [11].

We here analyzed the resulting N-glycan structures after the exposure of mouse lung endothelial cell cultures to constant and intermittent hyperoxia compared to control condition normoxia and hypoxia. We show that predominately sialylated and fucosylated N-glycan structures are transiently changed in response to high concentrations of oxygen. Under these oxygen conditions, the expression levels of certain enzymes in the biosynthetic pathway, including sialyl- and fucosyl- transferases, were changed in a time-dependent fashion. An intriguing question is, what consequences do such changes in the composition of the cell surface glycosylation have for the cell and for the organ lung?

Oxidative stress is known to have a huge general impact on endothelial function. A primary target is nitric oxide synthase, which, under oxidative stress, becomes uncoupled, resulting in NO deficiency and the generation of the aggressive reactive species peroxynitrite. This is, for instance, observed in smokers, as cigarette smoke contains a large amount of free radicals inducing oxidative stress and concomitant vascular diseases [12]. Other pathological conditions are associated with oxidative stress. For instance, in cancer hypoxia is frequently an important component that affects the cellular metabolism and results in altered cell surface expression including glycosylation. These alterations have an impact on disease progression with regard to angiogenesis and metastasis [13,14,15].

Cell surface glycosylation in general has an important function in providing structure with essential biophysical properties as well as presenting docking sites for proteins and cells. The presence or absence of distal sialic acids is a special important determinant and is dynamically regulated by the activity of sialic acid transferases and sialidases.

Mammalian sialyl-transferases comprise three families of 20 homologous enzymes that link sialic acids in α2-3, α2-6 or α2-8 linkage to terminal galactose or N-acetylgalactosamine of N- and O-glycans. Terminal sialic acids (frequently exhibiting further modifications, like different types of acetylation) provide cell surfaces with a protective electrostatic shield, which is generally anti-adhesive for circulating cells and protect cell surface glycoproteins from proteolytic attack [16,17].

In addition, sialic acids linked to glycoproteins and gangliosides are frequently recognized and used by pathogens, such as influenza and coronavirus (including MERS-CoV and SARS-CoV-2) to enter the host cells [18,19]. In the lung, it has been shown that sialylation of endothelial surface glycoproteins influences endothelial barrier function [13], and, as such, sialic acids are an important determinant of pulmonary endothelial barrier integrity.

Interestingly, endothelia from pulmonary artery endothelial cells (PAECs) and pulmonary microvascular endothelial cells (PMVECs) differ in the type of sialic acids especially with regard to their linkage. Neuraminidase treatment of these cell types from rat in vitro and in isolated perfused lungs has been shown to disrupt cell–cell and cell–matrix interaction [13]. In vitro, neuraminidase treatments led to the formation of intercellular gaps and modulation of impedance measurements and, in the perfused lungs, neuraminidase treatment induced pulmonary edema.

It can be speculated that the influence of the partial pressure of oxygen on endothelial cell surface sialylation can have an impact on lung barrier function and edema formation, which might be an important issue for ventilated ARDS patients treated with supplemental oxygen. It is still unknown, whether sialic acids are a structural component of the barrier integrity or mediate some kind of down-stream signaling that affects barrier function.

However, it has been shown recently that dependent on mechanical shear stress, sialic acids of the endothelial glycocalyx regulate the activity of the transcription factor erythroid 2-related factor 2 (Nrf2), which modulates antioxidant gene expression in the cell [20]. The authors show a direct link between surface sialic acids and the expression of VCAM-1, HO-1 and phosphorylation of eNOS, which are all important determinants of healthy vascular function. Our group has previously investigated the effect of hyperoxia in a static culture on these parameters, showing their dependence on oxygen condition and duration of exposure [21].

Glycans on the cell surface are frequently recognized in a sialic-acid pattern-dependent way by extracellular or cell-bound lectins, such as galectins (“galactoside binding lectins”) and siglecs (“sialic acid binding immunoglobulin-like lectins”) [22,23]. These interactions play an important role in the communication with the immune system.

Such questions can hardly be answered by using an in vitro model system, but nevertheless some basic mechanisms can be identified. In our experiments, we isolated the endothelial cells from whole mouse lungs, which will most likely represent a mixture of pulmonary artery and microvascular endothelial cells, although PMVECs can be assumed to predominate in numbers.

We observed a moderate shift of glycan structures in the course of cultivation without any further treatment, which is most likely due to de-differentiation of endothelial cells in culture. In order to minimize this problem, we repeatedly re-purified the cells using antibodies for endothelial markers to select for cells that maintain at least endothelial characteristics. In order to minimize culture-related bias, all exposures to hypoxic/hyperoxic conditions were compared to normoxia at the same time point from the same culture.

Under our experimental conditions, we found that, despite some functional overlap, distinct sialyltransferase subtypes were differentially regulated in their expression in response to different partial pressures of oxygen in a time-dependent manner. Changes in enzyme expression (i.e., early up-regulation of sialyl transferases under hyperoxia) go hand in hand to some extent with according structures of N-glycans as analyzed by mass spectrometry.

We observed a modest increase of complex sialylated N-glycans at 4 h exposure to strong hyperoxia that returned to baseline at later time points. It must be considered that not only N-glycans, but also other types of cell surface glycans are sialylated. We assessed global changes in sialylation by cell surface lectin binding but other N-glycan structures were not analyzed individually in this study.

Strong hyperoxia significantly increased expression of St6gal1 and St3gal2 at 4 h exposure. Sialyl transferase siRNA knockdown experiments revealed that St6gal1 is likely responsible for the increase of α2-6 sialic acid structures at the cell surface under hyperoxic conditions. St6gal1 is a sialyl transferase that adds α2-6 linked sialic acids to terminal N-glycans in glycoproteins and to glycolipids and has attracted significant attention as playing an important role in tumors.

Increased activation of this enzyme leads to remodeling of the endothelial cell surface glycome and concomitant altered recognition by lectins, such as galectins, which impacts the downstream signaling by cell surface receptors. Among others, especially effects on VEGF and PI3K/Akt signaling have been described [24]. The galectin 1-N-glycan axis further has an important role in regulating immune cell influx. In order to compare expression of sialyl transferases that prefer substrates other than N-glycans in the glycocalyx, we also looked at St3gal1 and St3gal2.

St3gal1 has a prime substrate specificity for type III glycans and forms Neu5Ac-α2-3Gal-ß1-3GalNc on mucin-type O-glycans (sialyl T-antigen) and gangliosides, St3gal2 is the main sialyl transferase responsible for ganglioside GD1 and GT1b biosynthesis. Expression of St3gal1 exhibits a similar response to different oxygen conditions over time as St6gal1. Interestingly, St3gal2 responds similar at early time points but has a huge increase in expression at 72 h under constant hyperoxia, pointing out the significant independent individual importance of the sialyl transferase family members.

Surface sialylation is not only regulated by Golgi-located sialyl transferases, but also by intracellular sialidases as well as plasma membrane and extracellular sialyl transferases and sialidases that remodel the de novo synthesized glycans and, therefore, provide a dynamic condition [25]. Sialylation is also frequently altered in disease states, like cardiovascular disease, COPD and cancer. A highly interesting discussion in the light of the ongoing COVID-19 pandemic centers around the question, whether these changes in sialylation might be responsible for the highly variable clinical manifestations of COVID-19, especially in the case of patients with comorbidities [26,27].

The virus-derived enzymes can interfere with the glycocalyx structure and also modify the interaction between sialylated cell surface glycans and binding partners, like siglecs, which can lead to exaggerated immune responses as observed in cytokine storms. As supplemental oxygen is in most cases vital for the treatment of severe COVID disease, it is of interest, whether oxygen-condition related changes in glycan structures could also have an impact on the clinical outcome.

Another interesting modification of N-glycans is fucosylation, which can occur on the innermost GlcNAc of N-glycans or in periphery. Changes in the expression levels of fucosyltransferases and, consequently, changes in the fucosylation patterns of glycans can contribute to the diagnosis of diseases and also to the monitoring of therapy success [28,29]. The α1-6 fucosyl transferase FUT8 adds fucose to the inner Asn-linked GlcNAc, and its state of activity plays an important role in the malignancy of cancer cells and metastatic potential.

A phenotype of FUT8 knock-out mice is at high risk of developing emphysema in the lungs. Interestingly, FUT8 expression was up-regulated early under strong and intermittent hyperoxia and again down-regulated at 24 h. An interesting expression pattern was observed for FUT3. FUT3 transfers fucose to a terminal GlcNAc creating the Lewis blood group antigens and after sialylation, the sialyl Lewis A or sialyl Lewis X epitope.

This plays an important role as tumor antigen or in lymphocyte homing, which is mediated by the interaction of L-selectin on lymphocytes and the carbohydrate ligand 6-sulfo sialyl Lewis X on high endothelial venules [30]. FUT3 was initially down-regulated at 4 h, but up-regulated at 24 h strong and intermittent hyperoxia. It remained upregulated under intermittent hyperoxia until 72 h, but was down-regulated under constant hyperoxia until 72 h.

Oxidative stress-related changes of protein glycosylation not only depend on the activity of glycan-modifying enzymes but also on the biosynthetic process in general. Under normal homeostatic conditions 2–5% of the total glucose metabolism is shunted into the hexosamine pathway (engaging the enzymes GFAT and UAP1), which generates as terminal product UDP-GlcNAc, a crucial substrate for N- and O-glycosylation, glycolipids, glucosaminoglycans as well as a precursor for sialic acids. Oxidative stress-related ROS generation inhibits the redox sensitive glycolytic enzyme glyceraldehyde-3-phosphate dehydrogenase (GAPDH) and increases the flux of glucose metabolites into the hexosamine biosynthetic pathway (HBP).

This occurs rapidly in the context of strong persistent hyperoxia reflected by an increase of total N-glycosylation after 4 h of constant 95% O_2_, which is again down-regulated at 24 h (including down-regulation of the rate-limiting HBP enzyme GFAT1) probably due to homeostatic compensation. In the long-term (72 h) protein expression of GFAT and UAP1 seem to be severely upregulated again. The activated HBP also spurs a special form of glycosylation, which is the attachment of a single GlcNAc to a serine or threonine by the enzyme OGT with the earlier mentioned impact on cellular signaling.

However, we did not observe a significant change of mRNA expression of OGT under constant or intermittent hyperoxia, but only a down-regulation of mRNA after 4 h and 72 h of constant hypoxia. It is known from the literature that hypoxia further downregulates OGT by proteasomal degradation [31].

Our experimental approach uses a cell culture model with all the advantages and drawbacks of an in vitro system: This comprises a purified cell population of lung endothelial origin, which allows to investigate direct cell-specific responses but is deprived of the in situ micro-environment and might undergo culture condition-dependent changes in phenotype.

In this study, we used three independent preparations from primary mouse endothelial cells, which were grown in vitro for almost 3 weeks. It is known that glycosylation patterns change depending on cell differentiation and also during in vitro cultivation, as recently also shown in a study on cardiomyocytes in culture [32]. Lung endothelial cells have been shown to be heterogenous per se.

Niethamer et al. used single cell analytics to characterize various different populations of pulmonary endothelial cells from mice, which have different gene expression and proliferation characteristics [33]. Primary lung endothelial cells are likely to change their phenotype in culture. This change in phenotype goes hand in hand with changes in glycosylation patterns, which might explain the high variability of quantitative results in our study. However, in most cases, the tendencies (up- or down-regulation) were comparable and consistent.

## 5. Conclusions and Perspective

Persistent hyperoxia induces a short-term disruption of homeostasis in N-glycosylation of lung endothelial cells (at 4 h exposure), which occurs as an increase in the general degree of total N-glycosylation and especially complex sialylated N-glycans. The effect is overcompensated thereafter, and baseline values are finally resumed until 72 h of exposure. Such transient changes in glycosylation can affect the down-stream signaling from cell surface receptors as well as interaction with blood proteins, cells of the immune system or pathogens as in the case of viral infections. Therefore, the impact of oxygen therapy on these processes might be of importance but is also difficult to delineate because of the complexity of putatively involved signaling networks in vivo.

Our data can only provide some basic mechanistic insights, which could support further investigations in whole animal studies or even clinical studies. No conclusions can be drawn at this point on which functional consequences these transient changes in endothelial glycosylation would have on the lungs. In the light of the ongoing pandemic, cell culture experiments could be further used to investigate, if the observed oxygen-dependent changes in cell surface sialylation have an influence for instance on the infectivity of SARS-CoV-2 or on disease progression. Experiments in small rodents could give insight into the effects on altered interactions with extracellular proteins or cells of the immune system.

Translation to clinical evaluation is generally rather complex, as standardized circumstances are very difficult in clinical settings with ventilated patients. Several studies have, for instance, shown acute degradation of the endothelial glycocalyx during heart surgery with cardiopulmonary bypass (CPB) [34,35]. Hyperoxia during CPB causes degradation of glycocalyx proteoglycans and aggravates ischemia/reperfusion injury of different organs [36,37]. Standardized clinical circumstances during heart surgery on CPB could be a possible approach to investigate the influence of altered partial pressures of oxygen on endothelial glycan structures and their impact on outcome.

## Figures and Tables

**Table 2:** Proteins identified in quantitative proteomic analysis as significantly changed in expression (Values: mean fold change relative to normoxia).

Exposure Condition	Protein	Fold Change Relative to 21% O_2_
24 h (0–95% O_2_) oscillation	Glutamine--fructose-6-phosphate aminotransferase [isomerizing] 1 (GFAT1)	0.3
24 h (0–95% O_2_) oscillation	Sialic acid synthase (N-acetylneuraminate-9-phosphate synthase) (NANS)	0.3
72 h (0–95% O_2_) oscillation	UDP-N-acetylhexosamine pyrophosphorylase-like protein 1 (UAP1)	2.3
72 h constant 95% O_2_	UDP-N-acetylhexosamine pyrophosphorylase-like protein 1 (UAP1)	2.2

## Data Availability

The authors confirm that the original/raw data supporting the findings of this study are available within the article and its Supplementary Materials.

## Supplementary Materials

The following are available online at https://www.mdpi.com/article/10.3390/antiox10121947/s1, (Supplemental Information S1A) High mannose N-glycan structures analyzed in this study (Supplemental Information S1B) Complex N-glycan structures analyzed in this study (Supplemental Data S2) Raw Data: Area under the curve of peaks from HPLC analysis (analysis January 2019, May 2019, August 2019). Mass lists: Glycan composition and derived monoisotopic mass. Retention times: Time in min. until glycan hit the detector. Analysis of degree of sialyltransferase knockdown 40 h after transfection with St6gal1 and St3gal6 siRNA by Western blotting (Supplemental Data S3).
