# Supplementary figures and images for: Oxygen-Dependent Changes in the N-Glycome of Murine Pulmonary Endothelial Cells
Source: Antioxidants (Basel). 2021 Dec 4;10(12):1947. doi: 10.3390/antiox10121947 (PMC8750181; doi:10.3390/antiox10121947)

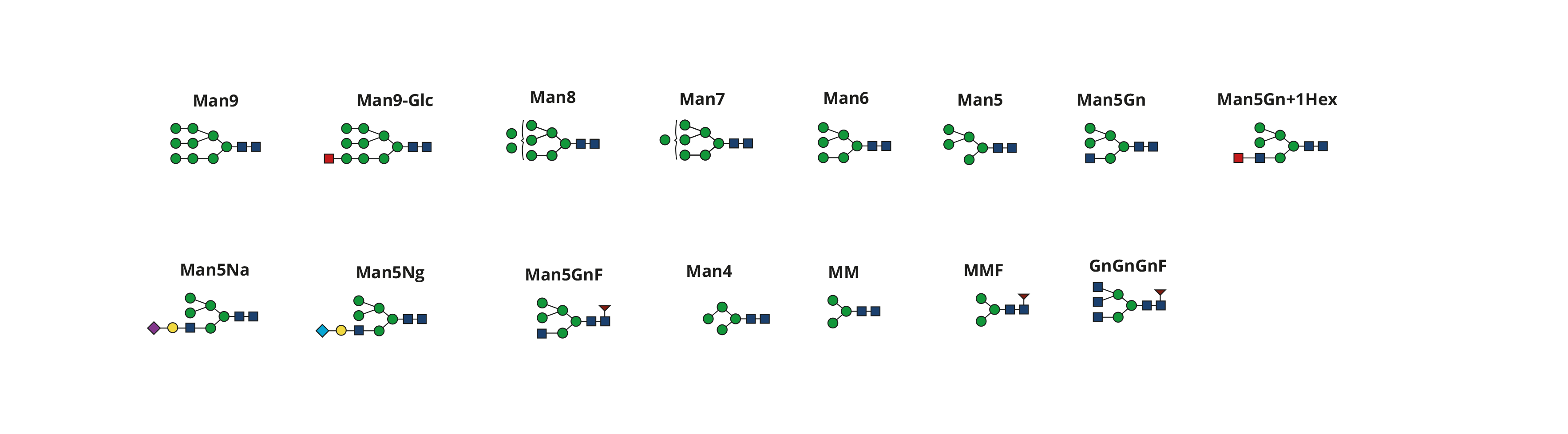

Supplement: Supplementary file 1 [file antioxidants-10-01947-s001.zip › Supplement S1A.tif]

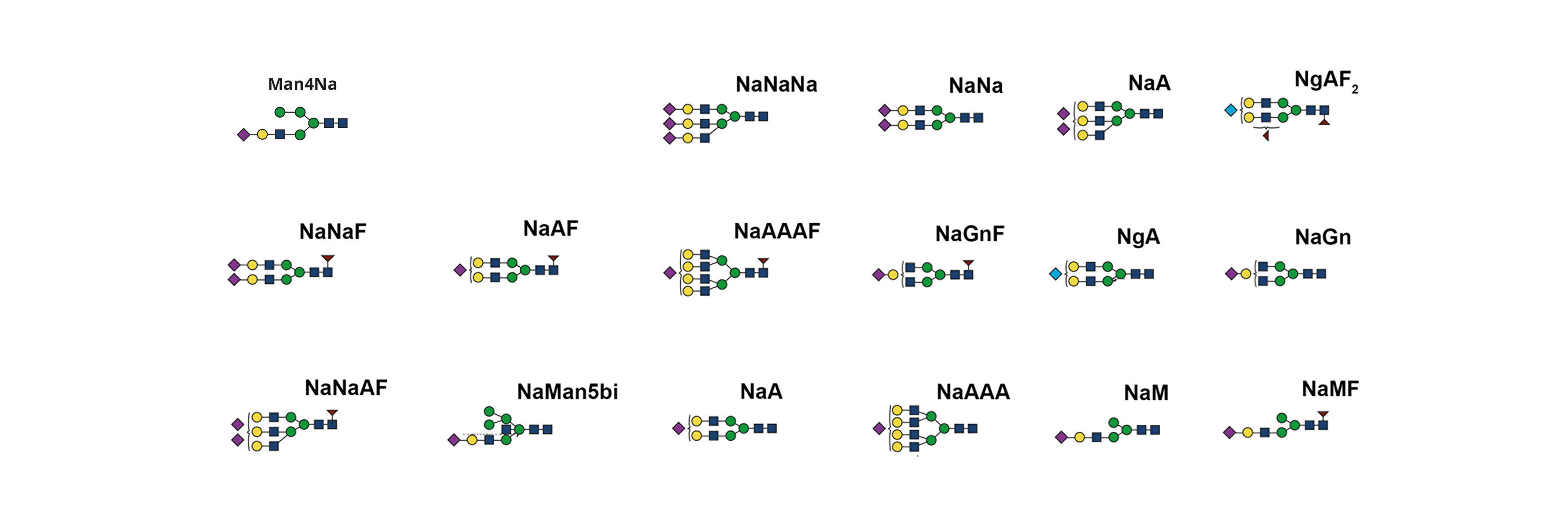

Supplement: Supplementary file 1 [file antioxidants-10-01947-s001.zip › Supplement S1B.tif]

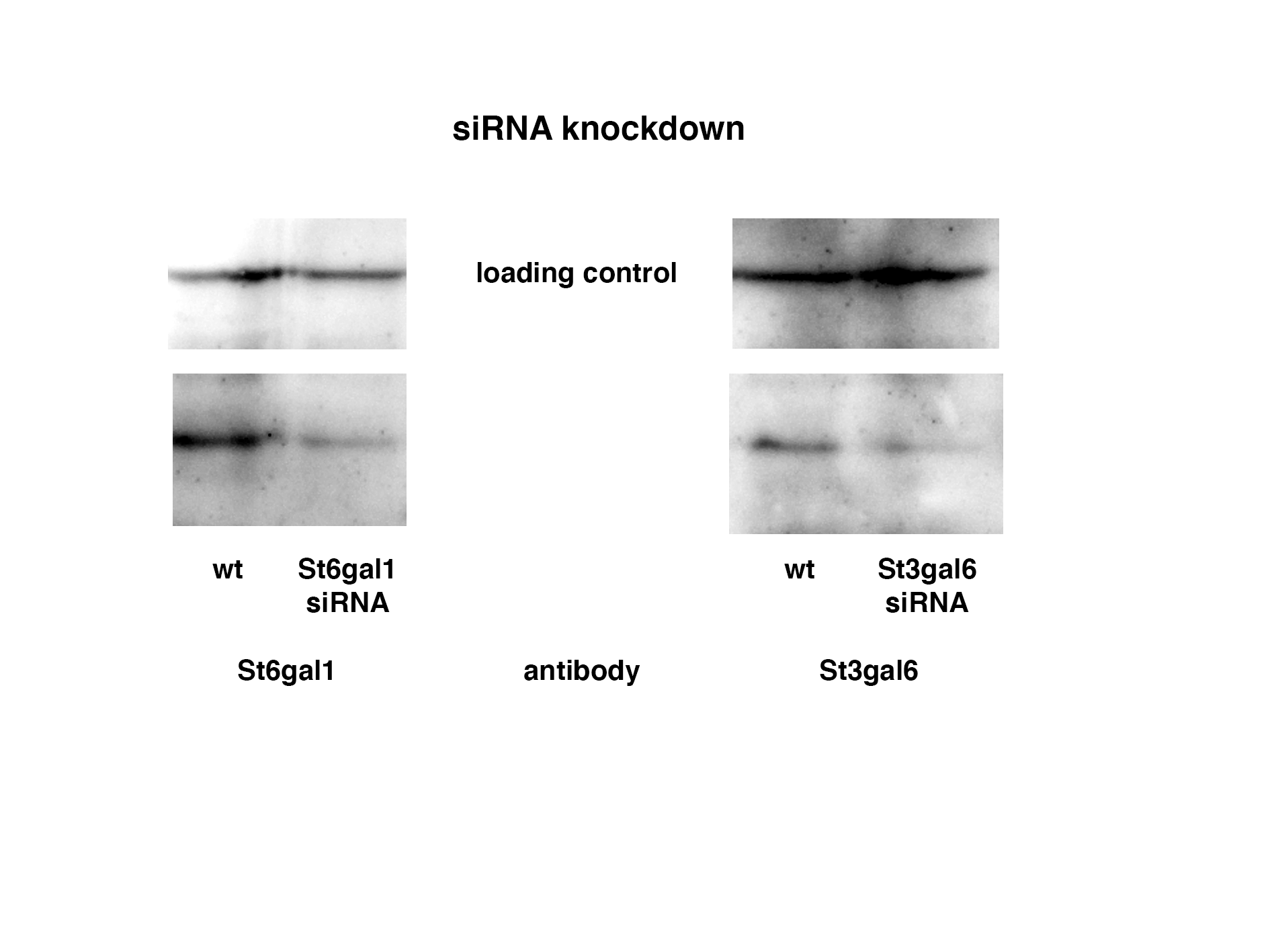

Supplement: Supplementary file 1 [file antioxidants-10-01947-s001.zip › Supplement S3.tif]
